# Bradycardia, Renal Failure, Atrioventricular-Nodal Blockade, Shock, and Hyperkalemia Syndrome: A Case Report

**DOI:** 10.7759/cureus.23486

**Published:** 2022-03-25

**Authors:** Arshan Khan, Abdelilah Lahmar, Moiz Ehtesham, Maria Riasat, Muhammad Haseeb

**Affiliations:** 1 Internal Medicine, Ascension St. John Hospital, Detroit, USA; 2 Medicine, Faculty of Medicine and Pharmacy/Mohammed VI University Hospital, Oujda, MAR; 3 Internal Medicine, Albany Medical Center, Albany, USA; 4 Internal Medicine, Icahn School of Medicine Mount Sinai Beth Israel, New York City, USA; 5 Internal Medicine, Bahria International Hospital, Lahore, PAK; 6 Internal Medicine, Jinnah Hospital Lahore, Lahore, PAK

**Keywords:** diagnosis of brash syndrome, pathophysiology of brash syndrome, management of brash syndrome, acute kidney injury and brash syndrome, brash syndrome

## Abstract

Bradycardia, renal failure, atrioventricular (AV) blockade, shock, and hyperkalemia (BRASH) syndrome is an uncommon and relatively new entity that results from synergy between AV nodal blockade and renal failure leading to a vicious cycle of hypotension, profound bradycardia, and hyperkalemia. Classically, this syndrome is seen in a patient taking AV nodal blocking agents and underlying renal insufficiency. We are presenting a case of a 76-year-old female with a medical history of essential hypertension and non-insulin-dependent type 2 diabetes mellitus presented to the emergency room with a chief complaint of dizziness and generalized weakness. The patient was taking metoprolol tartrate 200 mg twice a day, amlodipine 10 mg once daily, clonidine 0.1 mg twice daily, enalapril 20 mg twice daily, and Metformin 750 mg twice daily. On presentation, the patient had symptomatic bradycardia resistant to atropine with heart rate in 30s and hypotension resistant to volume expansion. The laboratory results showed that the patient also had acute kidney injury and severe resistant hyperkalemia. The whole presentation raised the suspicion of BRASH syndrome. The patient was started on peripheral dopamine infusion for bradycardia and symptomatic hypotension. Nephrology was consulted, and the patient was started on urgent dialysis for resistant hyperkalemia. The patient was admitted to the cardiovascular intensive care unit, and all antihypertensive medication, including beta-blockers, were stopped. The patient clinically improved on the next day, the dopamine infusion was stopped, and the patient remained vitally stable. The patient was eventually discharged home with cardiology and nephrology follow-up. The purpose of this case report is to help with the early diagnosis of this under-recognized and new clinical condition and to discuss the pathophysiology and management.

## Introduction

Bradycardia, renal failure, atrioventricular (AV) blockade, shock, and hyperkalemia (BRASH) are caused by a synergistic phenomenon that sets in motion a vicious cycle involving bradycardia, renal failure, atrial ventricular conduction blocking drugs, shock, and hyperkalemia [1]. Due to increasing comorbidities of the aging population requiring polypharmacy, this syndrome is becoming a more common cause of profound bradycardia [1]. Being a relatively newly identified entity, its diagnosis sometimes goes unrecognized, leading to the use of therapeutic models not based on the underlying pathophysiology, leading to an increase in patient morbidity and mortality [2]. The clinical manifestation varies from asymptomatic bradycardia to shock [1,2]. The therapeutic approach consists of basic supportive measures for hyperkalemia, bradycardia, and resuscitation with fluid therapy [3]. Herein, we present a case of BRASH syndrome with severe resistant hyperkalemia and bradycardia in a patient taking metoprolol tartrate.

## Case presentation

A 76-year-old female with a medical history of essential hypertension being treated with metoprolol 200 mg per os (PO) twice a day, amlodipine 10 mg PO once daily, clonidine 0.1 mg PO twice daily, enalapril 20 mg PO twice daily, and non-insulin-dependent type II diabetes mellitus being treated with metformin 750 mg twice daily was admitted to the emergency room by emergency medical services (EMS) with a chief complaint of dizziness and generalized weakness. The patient also reported a two-day history of watery diarrhea. The patient stated that she was regularly taking her medication as prescribed. On the day of admission to the emergency room, the patient felt weak and dizzy while in the restroom and fell to the ground. The patient reported hitting her head on the bathroom floor, but she denied loss of consciousness. The patient said that she was too weak to get up from the floor, so she stayed about three hours until her husband arrived home, and he called the EMS.

On EMS arrival, respiratory rate was 20 breaths/minute, SpO_2_ was 100% on room air, and heart rate was 30 beats/minute, with a blood pressure of 90/60 mmHg. EMS gave the patient 0.5 mg of IV atropine for bradycardia. Her respiratory rate was 16 breaths/min, SpO_2_ was 100% on room air, and her heart rate was 32 beats/minute, with a blood pressure of 73/45 mmHg. Her body temperature was 35.5°C, and her Glasgow Coma Scale (GCS) was 15. Her physical examination was otherwise unremarkable. 

**Figure 1:**
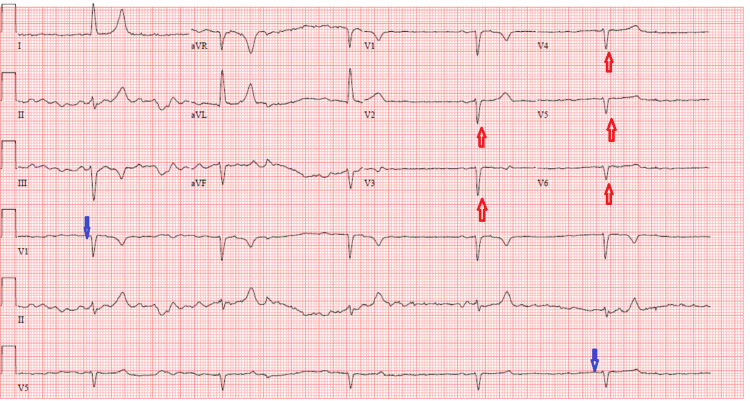
Admission ECG shows junctional rhythm with absent p waves (blue arrows), a heart rate of 32 beats/minute, and poor R-wave progression (red arrows).

The laboratory results on admission were as follows: Hemoglobin (9.2 g/dL), glucose random (117 mg/dL), sodium (128 mEQ/L), potassium (8.3 mEQ/L), chloride (99 mEQ/L), bicarbonate (10 mEQ/L), BUN (58 mg/dL), creatinine (6.2 mg/dL), lactic acid (5.2 mEQ/L) and anion gap of (19 mEQ/L). The patient's baseline creatinine was 1.0 mg/dL, and she had no known history of chronic kidney disease. A 12-lead electrocardiogram (ECG) showed junctional rhythm with absent p waves, a heart rate of 32 beats/minute, and poor R-wave progression (Figure 1).

The bradycardia, severe hyperkalemia, acute kidney injury, and hypotension while taking metoprolol tartrate raised the suspicion of BRASH syndrome. All antihypertensive medications were stopped. The patient was given 1 mg of intravenous atropine for symptomatic bradycardia, 5 mg of intramuscular glucagon two times, and a 3 L intravenous bolus of normal saline for hypotension. For severe hyperkalemia along with EKG changes, she received 10 mL of calcium gluconate (10%) intravenously over 2-3 minutes to stabilize cardiac myocytes, insulin 10 units and 50 mL of 50% dextrose three times, bicarbonate (8.4% W/V) 5 mL five times and nebulization with 20 mg of albuterol over 10 minutes three times. Patient's potassium was closely monitored every one hour after every treatment for hyperkalemia. Despite receiving multiple hyperkalemia treatments, the patient's potassium remained elevated at 7.2 mEQ/L. At that time, nephrology was consulted, and emergent hemodialysis was started. The patient remained bradycardic with minimal improvement in heart rate to as low as 40 s. She remained hypotensive with a blood pressure of 107/45 mmHg and a mean arterial pressure of 65.7 mmHg. She was started on dopamine infusion via a peripheral IV line. All antihypertensive and nephrotoxic medications were stopped. And the patient was transferred to the cardiac intensive care unit with close monitoring of vital signs and electrolytes.

An echocardiogram revealed an ejection fraction of 60% to 65% and grade 2 left ventricular diastolic impairment, otherwise unremarkable. Retroperitoneal ultrasound was obtained, which showed a nonobstructive left renal calculus, otherwise unremarkable. Urinalysis showed protein greater than 500, trace glucose, and trace ketones, otherwise unremarkable. The patient was successfully weaned off dopamine infusion and transferred to the general cardiac unit the next day. Her heart rate remained stable between 70 and 80 heartbeats per minute, and she did not require a pacemaker. The patient received two hemodialysis sessions consecutively on days 1 and 2 for hyperkalemia and acute kidney injury. After that, the patient did not require hemodialysis sessions as her hyperkalemia and creatinine started to improve without dialysis, and she started making urine. Later during the admission patient's bradycardia was resolved, and her blood pressure was running higher with an average blood pressure of 160/80 mmHg. So, patient home amlodipine 10 mg was resumed, and metoprolol tartrate was started at a lower dose of 75 mg twice a day by the cardiologist. On the day of discharge, the patient remained vitally stable; she denied any dizziness and fatigue. She remained in normal sinus rhythm without bradycardia on telemetry, and her serum creatinine was improved to 3.60 mg/dL and potassium of 4.0 mEQ/L. The enalapril was discontinued and after eight days of hospital admission, the patient was discharged with nephrology and cardiology follow-up.

## Discussion

Numerous cases have been published in the medical literature since the disease was codified in 2016 [3]. Still, it has not been recognized as a distinct entity, and little is known about its epidemiology [4]. Currently, the data used are from case reports showing a significant prevalence of underlying heart and kidney disease in the older adult population [5]. The syndrome is caused by a synergistic combination of hyperkalemia and AV node-blocking drugs that cause bradycardia [1]. Because bradycardia directly affects cardiac output, it can impede renal perfusion, leading to renal failure and aggravated hyperkalemia. This cycle, if left uncontrolled, can lead to multiple organ failure, including shock, bradycardia, and renal failure [4]. Mild clinical events can trigger the start of this cycle, such as dehydration, gastroenteritis, and poor oral intake, which have been implicated as precipitating factors leading to hypotension and prerenal azotemia [4,6]. It is worth noting that some drug interactions, such as angiotensin-converting enzyme inhibitors and angiotensin receptor blockers, may increase the risk of hyperkalemia and renal failure and thus contribute to the occurrence of BRASH syndrome, albeit these drugs are not essential to its development [4]. Our patient is diabetic and takes amlodipine, a calcium channel blocker. It has been suggested that nifedipine may cause bradycardia when the compensatory sympathetic drive is depressed, such as diabetes [7].

The clinical presentation of patients is highly variable; there are no specific symptoms or signs. It can range from asymptomatic bradycardia to cardiogenic shock [1,2]. A comprehensive clinical history with an emphasis on the history of pharmacological therapies, significant bradycardia, and hyperkalemia are critical elements in presenting a patient with BRASH syndrome. It should be noted that patients with BRASH syndrome usually present a clinical-biological incongruity. For example, ECG findings may range from junctional bradycardia to classic hyperkalemic changes, i.e., tall T waves, absent P and QRS widening even if the potassium level is mildly elevated [1-4]. Furthermore, there is disproportionality between the severity of bradycardia and AV nodal blocking drugs in the blood that is within the normal therapeutic range [3,4]. The serum potassium level in our patient was 8.3 mEq/L; her ECG findings display junctional bradycardia rhythm with poor P progression.

The diagnosis of BRASH syndrome is made mainly by clinical manifestations, ECG findings, and metabolic assessment after eliminating other possible responsible causes [3]. When encountering patients with refractory bradycardia, high clinical suspicion is required since the advanced cardiovascular life support (ACLS) bradycardia algorithm generally does not work for BRASH treatment [3], as in the case of our patient who was administered atropine via EMS received and when she was presented to the emergency room, her bradycardia was not appreciably improving and showed refraction from the standard treatment used in ACLS.

**Figure 2:**
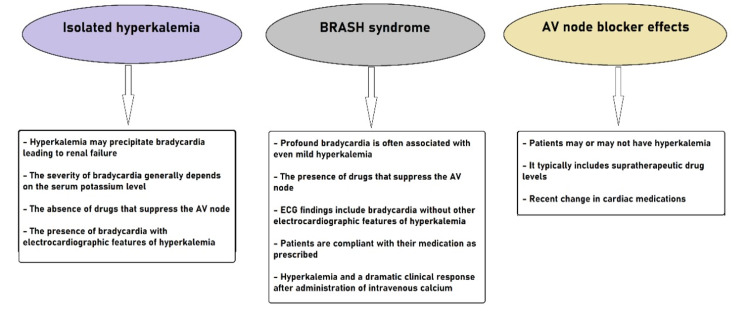
The key elements of clinical distinction between the differential diagnoses of BRASH syndrome Source [1,4]

Additionally, it is important to remember that due to the synergistic effect created by a combination of hyperkalemia and medications blocking the AV node, it might be difficult to distinguish between pure hyperkalemia and adverse effects due to AV node blocker. On the other hand, certain key indicators can guide the clinician in precisely defining the boundaries between these clinical entities [4]. Figure 2 schematizes the key elements for distinguishing between differential entities with BRASH syndrome.

Because this entity manifests as a syndrome with many signs, the clinician can focus their therapeutic efforts on implementing interventions that correct a single feature of the syndrome and overlook the entire clinical picture [1]. The application of the ACLS bradycardia management protocol, which has not provided good outcomes when used to treat the bradycardia of BRASH syndrome, is an example of this [8]. This underscores the need for a thorough understanding of the syndrome's pathophysiology. The use of atropine in bradycardia assumes that the origin comes from an effect mediated by the parasympathetic nervous system; however, the pathophysiology of BRASH syndrome is not related to the increase in parasympathetic tone, and as there is no expected response with atropine, the algorithm continues, resulting in the overuse of trans venous pacemaker [1].

Therefore, the approach aims to break the vicious circle by addressing the patient's conditions simultaneously. Treatment of hyperkalemia, even if minor, involves an intravenous administration of calcium, either calcium chloride or calcium gluconate; however, calcium gluconate is more tolerable for patients. Calcium gluconate is given in a typical dose of 10 mL of a 10% solution or 1 g IV and administered over 5-10 minutes [1-4]. The treatment can be repeated if the patient's ECG shows no improvement.

Insulin and dextrose should be administered intravenously to shift potassium intracellularly [4]. Nebulized albuterol can be given, potentially benefiting hyperkalemia and bradycardia [1]. In severe hyperkalemia, the use of antidiuretics in combination with high-dose loop diuretics and a thiazide diuretic is recommended, and patients with marked hyperkalemia and anuria often require emergency dialysis as definitive treatment for hyperkalemia [1,8]. Intravenous administration of calcium counteracts the cardiotoxic effects of hyperkalemia and improves bradycardia. In cases where the intended effect does not occur, epinephrine should be given, which increases the heart rate, increases systemic vascular resistance, and shifts potassium to the cells [1,4]. A push dose of 10 to 20 µg of a 1:100,000 dilution can be used to achieve this. [1,4,8]. Isoproterenol is a non-epinephrine chronotropic drug that may be effective in patients who do not respond to epinephrine [8]. Balanced solutions can be used in patients who are hypovolemic without acidosis. It is worth noting that normal saline should be avoided as it causes metabolic acidosis and a low anion gap that causes potassium to flow from intracellular to extracellular, worsening hyperkalemia [4,8,9]. Hypovolemia must be treated as soon as possible if present. Isotonic bicarbonate is often helpful for patients with uraemic acidosis and hyperkalemia [4].

The majority of mild BRASH syndrome cases respond favorably to basic medical intervention. Early detection of this disease improves prognosis and reduces the need for invasive procedures [1]. There are no specific guidelines about resuming AV nodal blocking agents after BRASH syndrome. The decision about continuing these agents once BRASH syndrome resolves can be made on a case-by-case basis. As in our patient, a cardiologist resumed the metoprolol at a low dose.

## Conclusions

The importance of BRASH syndrome stems from the fact that it is a life-threatening complication if treatment is not initiated promptly, as the cyclical nature of the syndrome rapidly leads to cardiac arrest, therefore requiring a high level of clinical suspicion. Despite the numerous cases reported in the literature, BRASH syndrome is currently a poorly diagnosed condition that can go completely unnoticed and requires knowledge of the syndrome and the physician's ability to identify patients where there is a risk that it will trigger some events. This involves a thorough examination of the pharmacological treatments used by patients to detect interactions.
